# LONG-TERM GENDERED PATHWAYS OF RELIGIOUS INVOLVEMENT POST WIDOWHOOD

**DOI:** 10.1093/geroni/igac059.1693

**Published:** 2022-12-20

**Authors:** Frances Hawes, Jane Tavares

**Affiliations:** University of Wisconsin-Eau Claire, Eau Claire, Wisconsin, United States; University of Massachusetts Boston, Boston, Massachusetts, United States

## Abstract

Widowhood is associated with decreased emotional well-being, particularly increased depression. Prior research suggests that religiosity may help improve mental health among widowed individuals. However, longitudinal studies exploring the role of religiosity on emotional well-being among widowed older adults is lacking, as are studies which examine different dimensions of religiosity. This longitudinal study analyzed data from the 2006-2018 waves of the nationally representative Health and Retirement Study (HRS). Ordinary least squares (OLS) regression analysis was used to examine the relationship between widowhood and depression as well as the role of religiosity as a moderator of this association. Analysis was stratified by gender to further explore these interactions. Results show that men and women show similar levels of depression at widowhood, but men are far less likely to be depressed prior to widowhood. Women also show a better recovery pattern over time post-widowhood. Furthermore, religiosity (particularly attending church) is an effective way of coping with widowhood and mitigating depression for both genders. However, men are significantly less religious than women. This study highlights the long-term effects of widowhood on depressive symptomology among older adults. Practical implications of this study include intervention development around increased screening and treatment for depression for widowed older adults (in particular, for widowers) as well as connecting this vulnerable population with resources. These findings may also inform program outreach (such as hospice bereavement services) that aim to facilitate healthy grieving among widowed older adults.

